# Leg and Joint Stiffness of the Supporting Leg during Side-Foot Kicking in Soccer Players with Chronic Ankle Instability

**DOI:** 10.3390/sports11110218

**Published:** 2023-11-07

**Authors:** Akihiro Tamura, Keita Shimura, Yuri Inoue

**Affiliations:** 1 Research Institute for Health and Sport Science, Nippon Sport Science University, Setagaya, Tokyo 158-8508, Japan; 2 School of Health Sciences, Tokyo International University, Kawagoe 350-1197, Saitama, Japan; kshimura@tiu.ac.jp; 3 Department of Physical Therapy, Faculty of Nursing and Rehabilitation, Konan Women’s University, Kobe 658-0001, Hyogo, Japan; y.inoue@konan-wu.ac.jp

**Keywords:** ankle sprain, joint instability, leg stiffness, joint stiffness

## Abstract

Soccer players with chronic ankle instability (CAI) may stabilize their supporting leg by the proximal joint to compensate for the ankle instability during kicking motion. This study aimed to investigate the characteristics of leg and joint stiffness of the supporting leg during side-foot kicking in soccer players with CAI. Twenty-four male collegiate-level soccer players with and without CAI participated in this study. The kinematic and kinetic data were obtained using a three-dimensional motion analysis system. Leg stiffness and joint (hip, knee, and ankle) stiffness in the sagittal and frontal planes were calculated and analyzed. The results clarified that soccer players with CAI (0.106 ± 0.053 Nm/°) had greater knee stiffness in knee adduction during the kicking cycle compared to those without CAI (0.066 ± 0.030 Nm/°; *p* = 0.046), whereas no characteristic differences were observed in knee stiffness in knee flexion and hip and ankle stiffness (*p* > 0.05). Knee stiffness is believed to occur to compensate for ankle joint instability in the supporting leg. Therefore, adjusting knee stiffness to accommodate ankle joint instability is crucial for maintaining kicking performance. Based on results of this study, it may be important to consider training and exercises focused on joint coordination to improve knee stiffness in soccer players with CAI.

## 1. Introduction

In soccer, musculoskeletal injuries occur frequently during games and practice [1]. Among these injuries, ankle sprains are the most common, accounting for 11% of the total injuries and 67% of all the ankle injuries in soccer players [2]. Ankle sprains often lead to persistent ankle instability, a pathological condition known “chronic ankle instability” (CAI) [3]. CAI is widespread and has two etiologies: mechanical ankle instability (MAI), defined as ankle movement beyond the physiological limit of the ankle range of motion [4], and functional ankle instability (FAI), defined as the subjective feeling of ankle instability and/or recurrent symptomatic ankle sprains due to proprioceptive and neuromuscular deficits [3,4]. These pathologies result from specific deficiencies in proprioception, neuromuscular control, postural control, strength, and other factors that affect the ankle complex mechanics [5].

A previous prospective cohort study found that 40% of patients with first-time ankle sprains developed CAI [6]. Therefore, it is essential to understand the causes of CAI after ankle sprain and how its modulation can affect injury prevention. Individuals with CAI exhibit postural control deficiencies and delayed muscle activation in the lower extremities [7]. Additionally, soccer players with CAI lack hip flexibility and have poor dynamic balance ability of the non-dominant leg, which is the supporting leg during kicking [8]. These findings suggest that soccer players with CAI may experience functional impairments and suboptimal dynamic alignment during motion. Recent systematic reviews have shown that CAI can impact lower extremity biomechanics during landings [9]. For instance, it can lead to increased knee flexion and reduced potential energy dissipation during side-cutting motions [10,11]. Moreover, due to compromised ankle sensorimotor function, individuals with CAI demonstrate tend to rely more on the hip joint for balance and stability during sports maneuvers [12]. These biomechanical findings highlight the necessity of assessing physical function and dynamic alignment in soccer players with CAI.

Kicking motions such as shooting and passing are integral to soccer games and have a significant effect on playing skills and performance [13]. Among these, side-foot kicking, in which the large medial area of the foot makes contact with the ball, is the most frequently used due to its power and accuracy. Successful side-foot kicking involves efficiently transferring energy from the supporting leg to the proximal-to-distal of the kicking-leg during the inside kick [14,15]. Recently, the supporting leg kinematics and kinetics of side-foot kicking have been noted as key factors in kicking performance and injury development [14,16,17]. A previous biomechanical study reported that the supporting leg generates a greater vertical ground reaction of 2.28 body weight during the initial heel strike in the instep kick [18]. Inoue et al. reported that knee extension of the supporting leg immediately before ball impact contributes to swing acceleration during kicking [14]. Furthermore, previous studies have suggested that the balancing ability and muscle strength of the supporting leg affect ball speed and kicking accuracy [19,20]. Therefore, maintaining adequate stability in the supporting leg is essential to achieve efficient energy transfer to the kicking leg.

Previous studies have suggested that CAI in soccer players is associated with poor dynamic balance of the supporting leg [8] and foot eversion during foot strikes [17], indicating potential difficulties in withstanding the impact during foot strike. However, individuals with CAI have greater knee stiffness during jump landings than those without, suggesting a possible compensatory mechanism for poor ankle joint stability and knee stiffness [21]. In addition, efficient kicking requires the transfer of energy from the ankle to the hip of the supporting leg, and then to the proximal-to-distal of the kicking-leg [14,15]. Therefore, soccer players with CAI may be required to maintain the entire stability of the supporting leg during side-foot kicking by increasing knee and hip stiffness of the supporting leg. To Identify stiffness properties of the lower extremities during kicking would provide a more comprehensive understanding of the ideal kicking motion for soccer players and would help to devise appropriate training methods. However, it is not clear whether soccer players with CAI can withstand impact during foot strike, that is, effectively maintain the stiffness of the supporting leg. This study aimed to investigate the stiffness characteristics of the supporting leg during side-foot kicking in soccer players with CAI. 

## 2. Materials and Methods

### 2.1. Participants

This study included 24 male collegiate-level soccer players (mean age, 20.8 ± 0.8 years; height, 172.4 ± 5.3 cm; body mass, 65.1 ± 5.3 kg) with a soccer playing experience of 10.9 ± 2.0 years. For all participants, the preferred leg for kicking (kicking leg) was the right leg, with the left leg acting as the supporting leg. Regarding their main position, 9 participants were strikers, 7 were midfielders, and 8 were defenders. The inclusion criteria were (a) age > 18 years and (b) >6 years of soccer experience. We excluded participants with (a) histories of orthopedic surgeries, (b) current injuries in the lower extremities, and (c) pain in the lower extremities during daily activities and soccer practice.

This study was conducted between August 2019 and March 2020 at the Kinematics Laboratory of the International University of Health and Welfare, Narita Campus, Chiba, Japan. 

Prior to testing, all participants volunteered to participate in this study and provided written informed consent. The study protocol was approved by the International University of Health and Welfare Ethics Committee (Approval No. 19-Io-59) and was conducted in accordance with the tenets of the Declaration of Helsinki [22].

### 2.2. Evaluation and Grouping of CAI

The participants completed a questionnaire regarding their physical characteristics, medical history of ankle sprains, and current medical information before inclusion in this study. The Cumberland Ankle Instability Tool (CAIT) was used to identify CAI [23]. The CAIT is a valid and reliable measure of the severity of FAI and is widely used by researchers and physicians [23,24,25], with a cutoff point of <27 [24,26]. 

The participants were divided into CAI and no-CAI (N-CAI) (CAIT score ≥ 28) groups. Those who had a CAIT score of ≤27 [23,26], a history of at least one significant ankle sprain, and a previously injured ankle joint “giving way,” recurrent sprain, and/or “feeling of instability” [27] were included in the N-CAI group.

### 2.3. Instrumentation

A three-dimensional motion analysis system (Vicon MX system, Oxford Metrics, Oxford, UK) and AMTI force plates with an amplifier (MSA-6 Mini Amp, AMTI, Watertown, MA, USA) were used to measure the kinetic data and GRF during testing, according to a previous study [17]. The sampling rate was set to 250 Hz for the three-dimensional motion analysis system and to 1000 Hz for the AMTI force plates.

All the participants wore closely fitted dark shorts, and the measurements were taken barefoot to accurately measure the data. Thirty-nine reflective markers (14 mm) were placed to create the coordinate systems of the head, torso, arm, pelvis, and leg segments, according to the Vicon Plug-in Gait full-body model (Oxford Metrics, Oxford, UK) [28]. Reflective markers were placed on the head (left/right front head and back head), the torso (7th cervical vertebrae, 10th thoracic vertebrae, clavicle, sternum, and right scapula), the upper arm and forearm (left/right acromion, upper arm, lateral epicondyle, forearm, radial styloid, ulnar styloid, and the head of the second metacarpal), the pelvis (left/right anterior superior iliac spine and posterior superior iliac spine), and the thigh and tibial segments (left/right lower lateral third of the thigh, lateral epicondyle, lower third of the shank, second metatarsal head, calcaneus) [17,28]. This segment model was used to create coordinate systems and to obtain the lower extremity kinematic and kinetic data.

### 2.4. Measurement Protocols 

Prior to testing, all the participants performed the desired warm-up, including slow jogging and/or stretching. Subsequently, the participants performed side-foot kicking trials on the AMTI force plates. A ball (Adidas Telstar 18; Adidas, Germany) was placed at the desired position on the AMTI force plates. All participants were instructed to perform side-foot kicking with as much force as possible toward a net (width and height 0.6 m each), positioned 2 m in front of the ball. The approach velocity was arbitrary for all participants. The approach angle was set at 45° with respect to the direction of the net. Side-foot kicking was conducted to obtain five successful trials. If the ball was off-target, the trial was excluded. The intervals between trials were set at 3 min to check the acquired data, reorganize the measurement settings, and consider the participant’s fatigue.

### 2.5. Data Analysis

The Vicon Nexus software (version 1.8.5; Oxford Metrics, Oxford, UK) was used to calculate all variable outcome measures based on the biomechanical model of the Vicon Plug-in Gait. The middle 3 trials of 5 successful kicking were used for data analysis. The vertical GRF, joint angles, and moments (the hip, knee, and ankle) of the supporting leg were obtained during a single side-foot kicking cycle. A single kicking cycle was defined as the duration from heel strike (when the vertical GRF first exceeded 10 N) to the frame in which the kicking leg reached its highest vertical position in the follow-through phase after ball contact. The kicking cycle was normalized as a percentage, that is, 0% and 100% represented the heel strike and frame of the highest vertical, respectively. 

Joint angles were derived using the Cardan y-x-z rotation sequence of the distal segment relative to that of the proximal segment. Positive joint angles represented flexion/dorsiflexion in the sagittal plane and abduction/inversion in the frontal plane. Joint moments were calculated as external moments; positive joint moments represented flexion/dorsiflexion in the sagittal plane and abduction/inversion in the frontal plane. The whole-body center of mass (CoM) was calculated from the weighted sum of all centers of mass of all segments defined by the markers. 

Leg stiffness (*K_leg_*) was calculated as the ratio of the peak vertical GRF (*vGRF_peak_*) to the change in vertical displacement of CoM (∆*L_CoM_*) [29], and formulated as Equation (1). *vGRF_peak_* was obtained from the maximum value during the kicking cycle. ∆L was calculated as the lowest and highest position of CoM during the kicking cycle. Leg stiffness (*K_leg_*) was normalized to the body mass (kg) and height (mm) of the participants

*K_leg_* = *vGRF_peak_*/∆*L_CoM_* (1)

Joint stiffness (*K_joint_*) of the lower extremities was calculated by the ratio of the change in joint moments (∆*M_peak_*) to the change in joint angles (∆*θ_joint_*) [29], and formulated as Equations (2)–(4). K_joint_ of the lower extremity joints were calculated as hip (*K_hip_*), knee (*K_knee_*), and ankle stiffness (*K_ankle_*) in the sagittal and frontal planes. ∆*M_joint_* represented the change in joint moments between the moment at the heel strike and the maximum value during the kicking cycle. ∆*θ_joint_* represented the angular displacement between the angle at the heel strike and the maximum value during the kicking cycle. Joint stiffness (*K_joint_*) of the lower extremities was normalized to the body mass (kg) and height (mm) of the participants

*K_hip_* = ∆*M_hip_*/∆*θ_hip_* (2)

*K_knee_* = ∆*M_knee_*/∆θ*_knee_* (3)

*K_ankle_* = ∆*M_ankle_*/∆θ*_ankle_* (4)

### 2.6. Statistical Analysis

Statistical analyses were conducted using the IBM^®^ SPSS^®^ Statistics for Windows version 27.0. (IBM Corporation, Armonk, NY, USA) by an independent statistician. Prior to statistical analysis, all outcome data were averaged for the middle three of five successful trials, and the means and standard deviations were calculated. Normality of data was assessed by the Shapiro–Wilk test, and homogeneity of variance was verified with Levene’s test. The independent sample *t*-tests were used to identify differences in variables between the CAI and N-CAI groups, depending on whether these variables were normally distributed. Statistical significance was set at *p* < 0.05. Effect sizes (ESs) were calculated using Cohen’s d from raw data by all outcomes. ESs are considered small when d = 0.20, medium when d = 0.50, and large when d = 0.80 [30]. Multiple regression analysis was performed to identify the contributors to leg stiffness by the angular displacement of the lower extremity joints during the kicking cycle. One-dimensional statistical parametric mapping (SPM[t]) unpaired *t*-tests were performed to compare each individual joint moment curve between groups using the open-source SPM1d code (www.spm1d.org, accessed on 22 March 2022) in MATLAB^®^ R2020b (The Mathworks Inc., Boston, MA, USA). 

## 3. Results

### 3.1. Partocipants Characteristics

Patient characteristics of both groups are shown in Table 1. There were no significant differences in the physical characteristics or playing experiences between the groups (*p* > 0.05). 

### 3.2. Joint Angles and Moments

**Figure 1:**
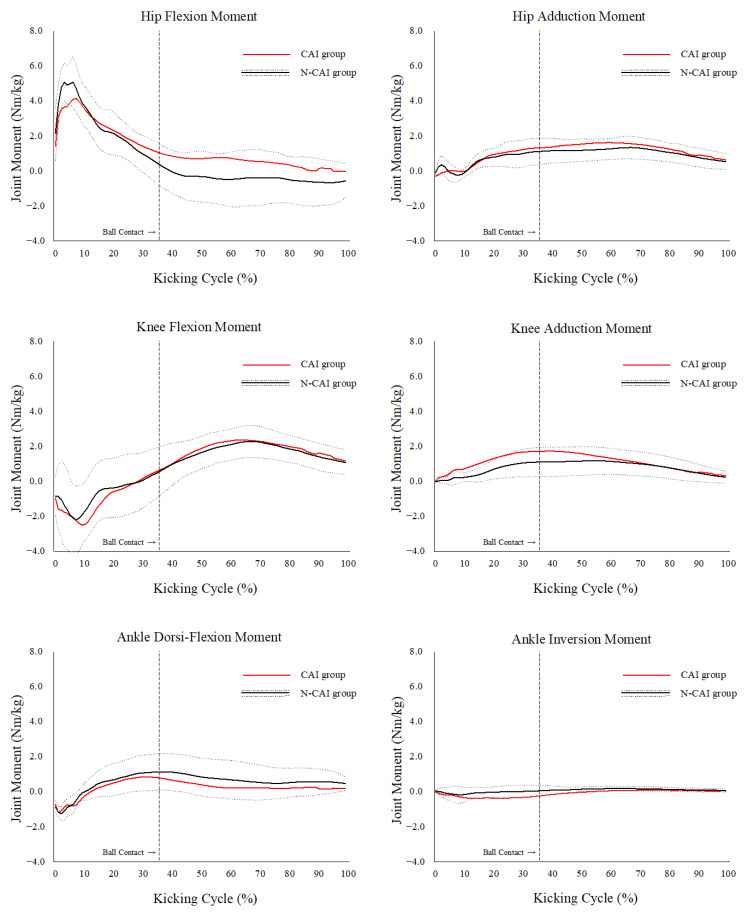
Time-series data of the hip, knee, and ankle joint moments of the supporting leg during the kicking cycle. The means and standard deviations of the CAI and N-CAI groups were expressed as red with the shaded areas and black lines with the diagonal lines. A kicking cycle was defined as the duration from the heel strike (0%) to the frame in which the kicking leg reached the highest vertical position in the follow-through phase after the ball contact (100%). CAI, chronic ankle instability; N-CAI, no-CAI.

The peak joint angles and moments of the supporting legs in the lower extremities are shown in Table 2 and Table 3. The peak knee adduction moment was significantly greater in the CAI group than that in the N-CAI group (1.30 ± 0.58 vs. 0.86 ± 0.39 Nm/kg, *p* = 0.049). There were no significant differences in the other variables during the kicking cycle between the CAI and N-CAI groups (*p* > 0.05). The joint moment curves of the CAI and non-CAI groups during the kicking cycle are shown in Figure 1. No between-group differences in the lower extremity joint moments were observed at any time point during the kicking cycle (*p* > 0.05).

### 3.3. Leg Stiffness

Leg stiffness did not differ significantly between the CAI (19.67 ± 2.85 kN/m) and N-CAI groups (22.21 ± 9.67 kN/m, *p* = 0.36, Table 4). The multiple regression model for the prediction of leg stiffness based on the angular displacement of the lower-extremity joints is presented in Table 5. Angular displacement in the sagittal plane was 32.9 ± 4.6° in hip flexion, 20.0 ± 3.6° in knee flexion, and 35.7 ± 7.0° in ankle dorsi-flexion. According to the multiple regression results, both angular displacements in the knee flexion and ankle dorsi-flexion were significant predictors of leg stiffness among the participants (R^2^ = 0.429, adjusted R^2^ = 0.344, *p* = 0.009).

### 3.4. Joint Stiffness of the Lower Extremity

The joint stiffness of the lower extremity joints is shown in Table 6. In the frontal plane analysis, the CAI group (0.106 ± 0.053 Nm/deg) showed significantly greater knee stiffness than the N-CAI group (0.066 ± 0.030 Nm/deg, *p* = 0.046), whereas there was no significant difference in the sagittal plane (*p* > 0.05). Regarding hip and ankle stiffness, these variables showed no significant differences in either the sagittal or frontal planes (*p* > 0.05). 

## 4. Discussion

This study aimed to investigate the stiffness characteristics of the supporting leg during side-foot kicking in soccer players with CAI. The results of this study showed that soccer players with CAI exhibit greater knee stiffness in the frontal plane during the kicking cycle compared to those without CAI; however, there were no characteristic differences in knee stiffness in the sagittal plane and hip and ankle stiffness. These results indicate that soccer players with CAI exhibit greater knee stiffness in knee adduction during kicking, potentially compensating for CAI-associated ankle instability.

Previous studies have revealed that leg stiffness is adjusted to accommodate different surfaces [31,32], increasing to accommodate reductions in surface stiffness. Thus, a reduction in surface stiffness results in increased joint stiffness of the lower extremities. The ankle, as the joint that absorbs the load after landing, plays a significant role in energy absorption [33,34,35], and therefore ankle instability causes poor alignment of the lower extremities during landing [36,37]. Li et al. reported that participants with CAI exhibit knee stiffness owing to ankle joint instability as a result of a protective strategy utilized to effectively absorb energy during landing [21], meaning that the stiffness and stability of the entire lower extremity may be maintained in legs with CAI. Using a similar mechanism, the soccer players with CAI in this study also had greater knee stiffness in the frontal plane during the kicking cycle and could compensate for ankle joint instability. Therefore, adjusting knee stiffness to accommodate ankle joint instability is considered to be very important for maintaining kicking performance, with properly managing CAI and learning the correct motion also being essential strategies. Previous studies have demonstrated that muscle recruitment can enable autonomously control joint stiffness, thereby enhancing dynamic joint stability by adjusting the joint stiffness [38,39]. Considering training and exercises focused on joint coordination may be useful to improving knee stiffness in soccer players with CAI.

Although the kicking motion was in the sagittal plane, the results of this study represented affected knee stiffness in the frontal plane. In a previous biomechanical study, the medial and lateral GRF components were generated in the supporting leg during the kicking cycle [17]. In addition, players with CAI had greater knee varus and hindfoot eversion angles at the flat-foot contact point [17]. This is because ankle instability in the frontal plane tends to be residual owing to anterior talofibular ligament sprain. Furthermore, this study showed that the peak knee adduction moment was significantly greater in the CAI group than in the N-CAI group. Therefore, players with CAI may be able to compensate for ankle joint instability by increasing knee stiffness in the frontal plane rather than in the sagittal plane. If knee stiffness cannot be increased to accommodate the ankle joint instability, it could lead to a reduction in the stiffness of the entire lower limb, possibly resulting in a lack of energy transfer in the kicking motion. 

There was no difference in leg stiffness, which represents stiffness of the entire lower extremity, between the CAI and N-CAI groups. This may be the result of ankle joint instability being compensated for by increased knee stiffness. Through the investigation of the angular displacements affecting leg stiffness among all players, it became clear that those at the hip and knee joints in the sagittal plane were the major contributors to leg stiffness. This suggests that knee and hip flexion are the most important factors in increasing leg stiffness during the kicking motion, similar to previous studies that investigated the main contributors of energy absorption during jump landing or side-stepping [34,40,41]. Therefore, it is important to recognize that knee and hip flexion are important factors that influence the stability of the supporting leg and efficiently transfer energy to the kicking leg during the kicking motion. 

Changes in the knee stiffness and kinematic/kinematic properties of the knee joint during landing motion are closely related to severe knee joint disorders such as anterior cruciate ligament injuries and knee osteoarthritis [42,43,44,45].In individuals with CAI, it has been reported that ankle instability with CAI is compensated for by increasing the knee stiffness with the less knee flexion, potentially resulting in inadequate energy attenuation and increased stress on the knee joint structures [9,46,47]. In addition, it has also been pointed out that increased knee adduction moment during landing, as in the results of this study, may lead to medial meniscus tears or future medial component knee osteoarthritis because of the large compressive load on medial tibial plateau due to forced knee varus [43,48]. Therefore, improvement of knee stiffness and a decrease in knee adduction moment would contribute to the prevention of secondary knee disorders. However, previous reports have been limited to biomechanical studies during jump landing and side-step movements, leaving unanswered questions regarding whether knee angular displacement in the sagittal plane or the knee stiffness in the frontal plane during kicking motion are associated with secondary severe knee injuries. Consequently, future research is warranted to determine if stiffness properties and kinematic/kinetics characteristics during kicking motion in soccer players with CAI are associated with future secondary disabilities.

This study had some limitations. Firstly, a previous study found that the CAIT measures the severity of CAI due to MAI, FAI, or a combination of these two phenomena [3]. As a result, CAIT scores may be affected by CAI severity and score. Hence, although the score has been validated as a reliable indicator for the severity of functional instability of the ankle, it should be considered as a self-reported evaluation tool and not equivalent to a physician’s evaluation. Secondly, kinematic and kinetic measurements with a multisegment foot model are hampered by measurement limitations and modeling assumptions [49,50]. All kicking trials were conducted barefoot to minimize measurement errors by shifting the reflective markers during kicking and in the laboratory, unlike in daily practice and games. Therefore, these results did not consider the effects of shock absorption by shoes or protection/fixation of the foot shape and the measurement environment differences from actual sports situations. Thirdly, this study had a small sample size per group after group categorization; therefore, it could be argued that the results might not provide accurate findings of the difference in stiffness properties between the CAI and Non-CAI groups. Considering the effects of the less sample size, we provided effect sizes for all the analyses to illustrate more meaningfully the magnitude of the differences between the groups. Primary outcomes resulted in a medium effect size of more than 0.3, which indicates that, despite the small sample size, our results provide a useful comparison of the differences in stiffness properties between groups.

## 5. Conclusions

The results of this study clarified that soccer players with CAI exhibited greater knee stiffness in the frontal plane of the supporting leg during the kicking cycle, while no characteristic differences were observed in the sagittal plane, compared to those without CAI. This finding indicated a potential link between ankle instability in CAI with knee stiffness of the supporting leg during side-foot kicking. Therefore, adjusting knee stiffness to accommodate ankle joint instability may be a crucial mechanism for maintaining kicking performance. These findings have relevance for the development of training programs and exercises centered around joint coordination to enhance knee stiffness in soccer players with CAI.

## Figures and Tables

**Table 1:** Physical characteristics of the participants in the CAI and N-CAI groups.

	CAI Group	N-CAI Group
N	14 (58%)	10 (42%)
Main position		
Striker, n	6	3
Midfielder, n	3	4
Defender, n	5	3
Age, years	20.8 ± 0.9	20.8 ± 0.8
Height, cm	172.8 ± 5.7	171.8 ± 5.0
Body mass, kg	65.8 ± 5.2	64.2 ± 5.6
Playing Experience, years	11.1 ± 1.7	10.6 ± 2.4
CAIT score	23.3 ± 4.1	29.6 ± 0.8

CAI, chronic ankle instability; N-CAI, no-CAI; CAIT, the Cumberland Ankle Instability Tool.

**Table 2:** Peak lower extremity joint angles in the sagittal and frontal planes of the CAI and N-CAI groups.

Variables	Groups	Mean ± SD ^1^	Mean Difference (95% CI ^2^)	*t* Value	*p* Value	Effect Size ^3^
Sagittal Plane						
Hip Flexion, deg	CAI	29.3 ± 9.4	−1.1 (−10.3–8.2)	−0.24	0.81	−0.10
	N-CAI	30.4 ± 12.5				
Knee Flexion, deg	CAI	33.3 ± 9.0	−3.4 (−10.5–3.6)	−1.01	0.32	−0.42
	N-CAI	36.7 ± 6.9				
Ankle Dorsi-Flexion, deg	CAI	23.9 ± 8.9	−0.7 (−7.3–5.9)	−0.23	0.82	−0.10
	N-CAI	24.6 ± 5.5				
Frontal Plane						
Hip Adduction, deg	CAI	8.3 ± 5.3	−2.0 (−5.8–1.8)	−1.09	0.29	−0.42
	N-CAI	10.3 ± 2.6				
Knee Adduction, deg	CAI	14.2 ± 13.7	7.9 (−2.8–18.5)	1.53	0.14	0.63
	N-CAI	6.3 ± 10.4				
Ankle Dorsi-Flexion, deg	CAI	6.3 ± 8.4	3.0 (−2.8–8.8)	1.09	0.29	0.45
	N-CAI	3.2 ± 3.3				

CAI, chronic ankle instability; N-CAI, no-CAI. ^1^ SD, standard deviation; ^2^ CI, confidence interval; ^3^ effect sizes were calculated using Cohen’s d.

**Table 3:** Peak lower extremity joint moments in the sagittal and frontal planes of the CAI and N-CAI groups.

Variables	Groups	Mean ± SD ^1^	Mean Difference (95% CI ^2^)	*t* Value	*p* Value	Effect Size ^3^
Sagittal Plane						
Hip Flexion, Nm/kg	CAI	1.60 ± 1.61	0.28 (−0.23–0.80)	1.14	0.27	0.47
	N-CAI	1.32 ± 0.58				
Knee Flexion, Nm/kg	CAI	1.44 ± 0.82	−0.37 (−1.20–0.45)	−0.94	0.36	−0.39
	N-CAI	1.81 ± 1.13				
Ankle Dorsi-Flexion, Nm/kg	CAI	2.54 ± 0.62	0.20 (−0.29–0.69)	0.84	0.41	0.35
	N-CAI	2.34 ± 0.49				
Frontal Plane						
Hip Adduction, Nm/kg	CAI	1.40 ± 0.63	0.01 (−0.46–0.48)	0.04	0.97	0.02
	N-CAI	1.39 ± 0.41				
Knee Adduction, Nm/kg	CAI	1.30 ± 0.58	0.44 (0.00–0.88)	2.09	0.049 *	0.86
	N-CAI	0.86 ± 0.39				
Ankle Dorsi-Flexion, Nm/kg	CAI	0.29 ± 0.37	0.04 (−0.24–0.31)	0.28	0.79	0.11
	N-CAI	0.25 ± 0.23				

CAI, chronic ankle instability; N-CAI, no-CAI. ^1^ SD, standard deviation; ^2^ CI, confidence interval; ^3^ effect sizes were calculated using Cohen’s d. * Statistically significant at *p* < 0.05.

**Table 4:** Leg stiffness of the lower extremity joints in the CAI and N-CAI groups.

Variables	Groups	Mean ± SD ^1^	Mean Difference (95% CI ^2^)	*t* Value	*p* Value	Effect Size ^3^
Leg stiffness, *K_leg_*, kN/m	CAI	19.67 ± 2.85	−2.52 (−8.16–3.11)	−0.80	0.36	−0.38
	N-CAI	22.21 ± 9.67				

CAI, chronic ankle instability; N-CAI, no-CAI^. 1^ SD; standard deviation, ^2^ CI; confidence interval, ^3^ effect sizes were calculated using Cohen’s d.

**Table 5:** Multiple regression analysis of leg stiffness and angular displacements in the sagittal plane.

Angular Displacements	Unstandardized Coefficients		Standardized Coefficients	*t* Value	*p* Value	95% CI ^1^
	B	Std. Error	Beta			
Hip Flexion, deg	0.17	0.26	0.14	0.66	0.52	−0.37–0.71
Knee Flexion, deg	−0.77	0.34	−0.48	−2.28	0.03 *	−1.49–0.07
Ankle Dorsi-Flexion, deg	−0.32	0.15	−0.39	−2.19	0.04 *	−0.63–0.02

^1^ CI, confidence interval; Std., standard; * statistically significant at *p* < 0.05.

**Table 6:** Joint stiffness of the lower extremity in the CAI and N-CAI groups.

Variables	Groups	Mean ± SD ^1^	Mean Difference (95% CI ^2^)	*t* Value	*p* Value	Effect Size ^3^
Sagittal plane						
Hip stiffness, *K_hip_*, Nm/deg	CAI	0.034 ± 0.012	−0.002 (−0.011–0.008)	−0.35	0.73	−0.14
	N-CAI	0.036 ± 0.008				
Knee stiffness, *K_knee_*, Nm/deg	CAI	0.061 ± 0.040	0.002 (−0.028–0.032)	0.16	0.88	0.06
	N-CAI	0.059 ± 0.025				
Ankle stiffness, *K_ankle_*, Nm/deg	CAI	0.037 ± 0.014	−0.003 (−0.013–0.008)	−0.56	0.58	−0.23
	N-CAI	0.039 ± 0.009				
Frontal plane						
Hip stiffness, *K_hip_*, Nm/deg	CAI	0.111 ± 0.058	0.017 (−0.026–0.060)	0.80	0.43	0.33
	N-CAI	0.095 ± 0.035				
Knee stiffness, *K_knee_*, Nm/deg	CAI	0.106 ± 0.053	0.040 (0.001–0.0780)	2.12	0.046 *	0.88
	N-CAI	0.066 ± 0.030				
Ankle stiffness, *K_ankle_*, Nm/deg	CAI	0.057 ± 0.045	0.014 (−0.019–0.046)	0.87	0.40	0.37
	N-CAI	0.043 ± 0.024				

CAI, chronic ankle instability; N-CAI, no-CAI^. 1^ SD; standard deviation, ^2^ CI; confidence interval, ^3^ effect sizes were calculated using Cohen’s d. * Statistically significant at *p* < 0.05.

## Data Availability

The data presented in this study are available on request from the corresponding author. The data are not publicly available due to privacy concerns.
